# Vascular Endothelium at the Molecular Level: From Fundamental Knowledge Toward Medical Implementation

**DOI:** 10.3390/biomedicines13010002

**Published:** 2024-12-24

**Authors:** Vladimir P. Shirinsky

**Affiliations:** Institute of Experimental Cardiology Named After Academician V.N. Smirnov, National Medical Research Center of Cardiology Named After Academician E.I. Chazov, Moscow 121552, Russia; shirinsky@gmail.com

The concept of multiple physiologic roles played by a single layer of endothelium on the intimal face of blood vessels started gaining recognition in the 1960s [1]. Since then, the understanding of molecular basis behind endothelial functions in an organism has increased remarkably and the translation of accumulated knowledge into novel diagnostic tools and treatment strategies to cope with multiple diseases involving the endothelium have begun. Given the ubiquitous presence of blood vessels in the human body, unsurprisingly, endothelium-oriented approaches may work for a wide range of pathologies, including cardiovascular and cerebrovascular diseases, cancer, diabetes, lung diseases, trauma conditions, infections, and many others.

By sensing blood flow as shear stress along its surface, the endothelium regulates arterial vessel diameter locally by delivering a proportioned blend of relaxing and contracting factors to the underlying smooth muscle cells in tunica media of resistive arteries. The most recognized of these factors are nitric oxide (NO) and endothelin-1 [2,3,4,5]. Furthermore, the blood flow-mediated regulation of vessel diameter does not require neural and hormonal activation and proceeds on its own as long as the heart pumps blood through the body, and the neuroendocrine control of vascular reactivity is critical for adjusting hemodynamic parameters for the needs of the organism as a whole. Additionally, angiotensin-converting enzymes ACE and ACE2 on the surface of endothelial cells produce angiotensin-II and angiotensin (1–7) peptides, respectively, from the angiotensin-I precursor when the latter is available through kidney-derived renin activity. Angiotensin-II augments the contractile response of vascular smooth muscle cells, while vasorelaxing angiotensin (1–7) counterbalances this activity through the G-protein-coupled MAS receptor [6,7,8]. An imbalance in signals from the endothelium to arterial smooth muscle cells causes excessive contraction of the arterial wall that is further exacerbated when arteries lose elasticity and blood pressure is permanently elevated, thus contributing to the development of arterial hypertension.

Based on molecular findings, a plausible approach in the treatment of arterial hypertension would be to restore NO/prostacyclin/EDHF production by vascular endothelial cells. This idea has been up in the air for a long time [9,10]. However, no drugs except ACE inhibitors currently prescribed to hypertensive patients achieve this goal. They primarily target vascular smooth muscles, the kidneys, and the central nervous system [11]. Moreover, angiotensin-II receptor blockers and beta-adrenoreceptor blockers, in fact, suppress the natural vasorelaxing activity of the endothelium when they target corresponding receptors on endothelial cells [12,13]. Although these pharmaceuticals are effective in blood pressure reduction, the future emergence of endothelium-oriented hypotensive medicines that restore the natural balance of relaxing/contracting/extracellular matrix cross-linking activities may profoundly affect the field of arterial hypertension treatment.

An intact endothelium is athrombogenic and prevents platelet adhesion and clot formation. This is, in part, due to glycocalyx being present on the luminal surface of endothelial cells. Besides serving as an amplifier for the shear stress sensors of the endothelium, this fine-mesh and hair-like structure with a prominent negative charge repels platelets as well as red blood cells from the vascular wall [14]. Synergistically with glycocalyx, the continuous release of prostacyclin and NO by the endothelium into blood keeps platelets quiescent through intraplatelet signaling by cyclic nucleotide-activated protein kinases PKA and PKG [10,15]. The heparin-like sulfated components of endothelial glycocalyx provide attachment sites for liver-derived antithrombin III that binds and inactivates thrombin and several other clotting factors. Additionally, endothelial cells expose thrombomodulin on their surface, which also binds thrombin and switches its activity from being procoagulant to anticoagulant through protein C activation. Besides these anticoagulation mechanisms, endothelium secrets tissue factor pathway inhibitors (TFPIs) as well as plasminogen activators t-PA and u-PA, which stimulate fibrinolysis [16].

On the other hand, the endothelium produces thromboxane A2 and PAF, potent platelet activators [17,18], as well as plasminogen activator inhibitor PAI-1, which suppresses fibrinolysis [16]. The endothelium is the principal storage site of von Willebrand Factor (vWF), which critically important for hemostasis [19]. Importantly, vWF activates at high shear rates of blood flow when its globular multimers unfold into strands and efficiently interact with collagen on endothelium-denuded vascular walls and with platelets [20]. Such high shear rates take place in areas of arterial injury and bleeding. Hemostasis in this situation is vital for the organism. Similar excessive shear rate conditions occur in areas of atherothrombosis, where the endothelium is unable to adequately adjust vessel diameter and reduce the blood flow velocity. Blood coagulation in this case proceeds similarly to during bleeding; however, the outcome is the opposite. It creates a threat to the organism by interrupting blood supply to downstream tissues.

Overall, the vascular endothelium is actively involved in hemostatic and thrombotic events. Still, contemporary antithrombotic therapies target platelets and blood clotting factors. These drugs are effective, although some of them, such as COX-1 inhibitor aspirin, in fact causes endothelial dysfunction by inhibiting prostaglandin synthesis [21]. Since many key molecular participants of blood coagulation on the endothelial side are identified, there seems sufficient grounds to consider endothelium-targeted approaches in antithrombotic pharmacotherapy that may bring this field to a new level of efficacy. Protecting endothelial glycocalyx is one such option worth investing effort into.

The endothelium is an indispensable player in inflammatory reactions and immune defense. Various proinflammatory agents (histamine, PAF, IL-1, TNFα, LPS, etc.) activate endothelial cells to expose E- and P-selectin, ICAM-1/2, and VCAM-1 adhesion molecules on their surface [22,23]. They attract and tether leukocytes from circulation and accumulate them at the sites of infection. Another key endothelial reaction during pathogen intrusion is to seal this area away from healthy tissue by increasing microvascular permeability and forming a local edema. This obstructs the surrounding blood vessels and prevents dissemination of the infection, as well as sustains high levels of bactericidal substances generated by leukocytes in the inflammation core. When this inflammation is resolved, blood supply is restored to the area.

Unfortunately, the same hyperpermeability reaction by the microvascular endothelium becomes dangerous when it becomes excessive and generalized and develops in critical organs such as the brain and lungs. Multiple insults in addition to infection, including chemical, hormonal, hypoxic, and reperfusion stress or excessive physical deformation, compromise the microvascular barrier’s integrity and lead to protein-rich tissue edema [24]. The acute development of edema of the lungs or brain presents serious medical challenges yet remains a pharmacologically unmet urgent condition that quickly transforms into multiorgan failure associated with high mortality. Currently, the use of osmotic agents, steroids, and diuretics in critical edema cases collectively accounts for about 40% of patient survival at best [25,26,27].

Pharmacologic developments in this highly important problem area have not yet resulted in clinically approved treatments and largely remain at the basic research/preclinical levels. It is now established that endothelial cells attenuate cell–cell contact in response to stress through multiple signaling events involving protein phosphorylation–dephosphorylation as fast-response (minutes) regulatory circuits [28]. Notable progress has also been made in understanding the molecular arrangement of these circuits. Key protein kinases and protein phosphatases and their target cytoskeletal/motor and intercellular contact proteins have been identified [29]. One of the principal determinants of endothelial hyperpermeability and a viable molecular target for antiedemic drug development is endothelial 210 kDa myosin light chain kinase [30,31]. Several signaling pathways have been discovered that strengthen the endothelial barrier. Natural substances activating these pathways include sphingosine-1-phosphate, angiopoietin-1, adrenomedullin, catecholamines, ATP, and NO [32]. Due to pleiotropic effects and stability issues, these substances require further modification/optimization before they can be used to target the endothelium in patients with acute edema.

The microvascular endothelium is the gatekeeper in multiple metabolic pathways. The semipermeable endothelial barrier between blood and tissues delivers nutrients and oxygen to the surrounding cells while accepting carbon dioxide and slag substances back in the blood for further utilization in the lungs, liver, and kidneys. The barrier capacity depends on the performance of the inter-endothelial contact and endothelial cytoskeleton/contractile machinery as well as on the transport through the endothelial cells. Water-soluble substances with a molecular radius less than 3 nm such as glucose, urea, ions, etc. freely diffuse through the junctions between the endothelial cells in a monolayer, whereas traffic from larger molecules such as peptide hormones and proteins employ highly regulated vesicular transport [24]. Although small in size, hydrophobic free fatty acids (FFAs)—the main energy source for skeletal muscles and the heart—require protein carriers to cross the endothelial monolayer [33]. FFAs are transported through the endothelial cell body. Endothelial glycocalyx is also viewed as a part of the barrier that can discriminate the passage of compounds based on their charge [14]. The contribution of the extracellular matrix beneath the endothelial cells (basement membrane) to the total barrier was estimated at 50% based on in vitro experiments [34].

The microvascular endothelium is functionally tuned to daily fluctuations in glucose and FFA levels that synchronize with fasting and postprandial periods. However, persistent metabolic stress causes endothelial dysfunction and further demise [35]. The endothelium is exposed to hyperglycemia and hyper/dyslipidemia in patients with type 2 diabetes mellitus, a widespread pathology prevalent among all diabetes cases. Persistent hyper/dyslipidemia at the endothelial face is also typical for atherosclerosis. Clinical observations attest to the fact that atherosclerosis starts earlier and is more severe in patients with diabetes than without this comorbidity [36]. This suggests that high blood glucose predisposes and, in combination with lipid misbalance, damages the vasculature starting at the endothelium. Of note, a recent report demonstrated that long-term exposure of human endothelial cells to isolated 30 mM glucose (5-fold excess over normal glucose levels in the human plasma) does not suffice to impair the endothelial barrier integrity and function in vitro [37]. Thus, how chronic exposure of endothelial cells to elevated glucose affects their lipid tolerance and overall ability to resist metabolic stress needs to be established at the molecular level. Among the possible candidates in this search is AMP-activated protein kinase (AMPK), the key energy sensor, metabolic regulator, and survival agent of the cell [38]. Another candidate is pulsatile flow/shear stress, which continuously affects the endothelium in vivo and activates the number of known and, perhaps, yet unknown signaling cascades promoting cell survival. Based on future findings in these and other directions, novel approaches for vascular protection in diabetes and cardiovascular diseases could evolve.

Endothelial cells and their progenitors are responsible for wiring developing organisms with blood vessels. They achieve this through the processes of vasculogenesis (de novo vessel formation) and angiogenesis (vascular branching from preexisting vessels). In adult organisms, angiogenesis prevails and may be initiated by tissue hypoxia. Hypoxia-induced factor 1α (HIF-1α) is stabilized in oxygen-starving cells and, in complex with HIF-1β, drives the expression of the vascular endothelial growth factor (VEGF) gene [39]. The VEGF gradient is the main angiogenic signal that stimulates endothelial cell proliferation; chemotaxis; and the formation of novel, though leaky, vessels. Other signaling molecules such as angiopoietin-1 are required to enhance the inter-endothelial contact and strengthen the endothelial barrier of new vessels [40]. Through an angiogenic reaction of the endothelium, collateral blood circulation in the regions that suffer reduced blood supply due to atherothrombosis/vascular obliteration can be established.

Intensive research efforts toward understanding the molecular mechanisms of angiogenesis resulted in therapeutic angiogenesis strategies that include the use of minigenes of the VEGF, HGF, and FGF growth factors encoded in plasmid DNA or packed in viral vectors; growth factors as proteins; and partially differentiated cells as emitters of a blend of angiogenic factors [41]. However, the transfer of angiogenic technologies to clinical practice is slow due to the controversial/limited results of clinical trials and the high costs of gene-based therapeutic angiogenesis. Currently, plasmid DNA-encoded VEGF (Neovasculgen) is approved in Russia and Ukraine, and HGF (Collategene) is approved in Japan. For patients with severe ischemic conditions in other parts of the world, these angiogenic drugs are not yet available.

At the same time, there are serious medical conditions when angiogenesis has to be halted to block disease progression. Based on Folkman’s inspiring idea that solid tumors rely on angiogenic factors for building tumor vasculature [42], antiangiogenic therapy is now widely used in oncology and beyond. Diabetic retinopathy results in blindness due to excessive VEGF-dependent local microvascular formation accompanied by retinal edema and vitreous hemorrhages. The inhibition of local angiogenesis is considered in wet macular degeneration, rheumatoid arthritis, psoriasis, and possibly atherosclerosis. To reduce systemic or local levels of VEGF in patients anti-VEGF antibodies are used. Bevacizumab was the first VEGF-targeted humanized monoclonal antibody approved by the FDA in 2004. Other clinically approved antiangiogenic treatments include antibodies to VEGF receptors and multiple receptor tyrosine kinase inhibitors that block signaling by VEGF and other growth factors by targeting the cytoplasmic protein kinase domains of the growth factor receptors on endothelial cells [43].

Situations in which pro- and antiangiogenic strategies are achieved within the same organism are easy to imagine. However, ideally, neither strategy should interfere with the other. Precise targeting of antiangiogenic agents to solid tumors is one of the options worth developing. Further insight into the molecular mechanisms behind angiogenesis/antiangiogenesis regulation is anticipated to identify novel components suitable for translation into the clinical setting to achieve effective negative as well as highly needed positive control of vascular growth.

The contributions to the current Special Issue address the various molecular aspects of endothelial functions briefly outlined above. Fundamental topics such as actin cytoskeleton and protein phosphatase participation in barrier regulation are accompanied by preclinical/clinical assessments of molecular data on glycocalyx, endothelin-1, endothelium-derived relaxing factors, and cross-linking factors such as lysyl oxidase. The role of succinate, a Krebs cycle metabolite, in the metabolic/non-metabolic regulation of angiogenesis is also reviewed. In addition, new results on the role of AMPK isoforms and pulsatile flow in protecting endothelial cells from metabolic stress exerted by pathologic FFA concentrations are presented.

As a Guest Editor of this Special Issue, I am grateful to all the contributors for providing a multiangle view of the current developments and future directions in the field of vascular endothelium research at the molecular level.
